# Supplementary material for: Increased Temperature Effects During Fruit Growth and Maturation on the Fruit Quality, Sensory and Antioxidant Properties of Raspberry (Rubus idaeus L.) cv. Heritage
Source: Foods. 2025 Mar 29;14(7):1201. doi: 10.3390/foods14071201 (PMC11988984; doi:10.3390/foods14071201)
Supplement: Supplementary file 1 [file foods-14-01201-s001.zip › foods-3545317-supplementary.pdf]

Supplemental Table S1. Soil analysis and nutrient composition of central orchard during 2024 (Casablanca).

UNIVERSIDAD AUSTRAL DE CHILE
Faculty of Agricultural and Food Sciences
Horticulture Soil Laboratory
Analysis: Soil Chemical - Soil Physical - Foliar - Fertilizers - Waters

GROWER ID			
Name	24/959		
Location	Casablanca		
Income	01,03,2024	Delivery	22,03,2024

Identification								
Laboratory number			24/959					
Soil			Raspberry cv. Heritage Soil					
Depth		(cm)	0-30					
**Results**								
**pH**	In water (1:2,5)		7,2					
**pH**	$CaCl_2$ (0,01M) (1:2,5)		6,4					
**Organic Matter**		(%)	1,1					
**N- Mineral**	($N\text{-}NO_3+NH_4$)	(mg/kg)	11,2					
**Phosphorus (P)**	Olsen	(mg/kg)	140,5					
**Potassium (K)**	exchangeable	(mg/kg)	579					
**Sodium (Na)**	exchangeable	($cmol^+/kg$)	0,05					
**Calcium (Ca)**	exchangeable	($cmol^+/kg$)	5,57					
**Magnesium (Mg)**	exchangeable	($cmol^+/kg$)	1,97					
**Bases**	exchangeable	($cmol^+/kg$)	9,07					
**Aluminum**	exchangeable	($cmol^+/kg$)	0,01					
**CICE**		($cmol^+/kg$)	9,08					
**Aluminum saturation**		(%)	0,1					
**Sulfur (S)**	available	(mg/kg)	2,0					
**Boron (B)**	available	(mg/kg)	0,86					
**Zinc (Zn)**	available	(mg/kg)	4,06					
**Iron (Fe)**	available	(mg/kg)	21,7					
**Copper (Cu)**	available	(mg/kg)	2,19					
**Manganese (Mn)**	available	(mg/kg)	14,1					
**Aluminum**	extractable	(mg/kg)	11					

Note: The soil analytical procedures used by our laboratory are those recommended by the Chilean Society of Soil Science and the National Accreditation Commission (CNA).

FERNANDO RODRIGUEZ SOTO
Agronomist
Head of Soil Analysis Service

Laboratorio de Suelos de Agronomía
Edificio C 2º piso - Campus Isla Teja - Casilla 567 - Valdivia - Chile
Fono 63-221239 - 293123 Fax 63-221430 E-mail labsuelos@uach.cl - analisisdesuelos@uach.cl

Supplemental Table S2. Soil analysis and nutrient composition of southern orchard during 2024 (Paillaco).

UNIVERSIDAD AUSTRAL DE CHILE
Faculty of Agricultural and Food Sciences
Horticulture Soil Laboratory
Analysis: Soil Chemical - Soil Physical - Foliar - Fertilizers - Waters

GROWER ID		
Name	24/1218	
Location	Paillaco	
Sample arrival:	01,03,2024	Delivery 22,03,2024

ORCHARD ID								
**Identification**			24/1218					
Laboratory number			Paillaco					
Soil		(cm)	0-20					
Depth		(ha)						
**ANALYTICAL RESULTS**								
**pH**	In water (1:2,5)		5,8					
**pH**	$CaCl_2$ (0,01M) (1:2,5)		5,3					
**Organic Matter**		(%)	12,7					
**N- Mineral**	$(N\text{-}NO_3+NH_4)$	(mg/kg)	16,1					
**Phosphorus (P)**	Olsen	(mg/kg)	15,9					
**Potassium (K)**	exchangeable	(mg/kg)	165					
**Sodium (Na)**	exchangeable	($cmol^+$/kg)	0,05					
**Calcium (Ca)**	exchangeable	($cmol^+$/kg)	5,57					
**Magnesium (Mg)**	exchangeable	($cmol^+$/kg)	0,92					
**Bases**	exchangeable	($cmol^+$/kg)	6,96					
**Aluminum**	exchangeable	($cmol^+$/kg)	0,03					
**CICE**		($cmol^+$/kg)	7,00					
**Aluminum saturation**		(%)	0,5					
**Sulfur (S)**	available	(mg/kg)	14,7					
**Boron (B)**	available	(mg/kg)	0,21					
**Zinc (Zn)**	available	(mg/kg)	0,19					
**Iron (Fe)**	available	(mg/kg)	26,1					
**Copper (Cu)**	available	(mg/kg)	0,29					
**Manganese (Mn)**	available	(mg/kg)	2,30					
**Aluminum**	extractable	(mg/kg)	1.168					

Note: The soil analytical procedures used by our laboratory are those recommended by the Chilean Society of Soil Science and the National Accreditation Commission (CNA).

FERNANDO RODRIGUEZ SOTO
Agronomist
Head of Soil Analysis Service

Laboratorio de Suelos de Agronomía
Edificio C 2º piso - Campus Isla Teja - Casilla 567 - Valdivia - Chile
Fono 63-221239 - 293123 Fax 63-221430 E-mail labsuelos@uach.cl - analisisdesuelos@uach.cl
